# Brain structural and functional anomalies associated with simultanagnosia in patients with posterior cortical atrophy

**DOI:** 10.1007/s11682-021-00568-8

**Published:** 2021-11-17

**Authors:** Yue Cui, Yang Liu, Caishui Yang, Chunlei Cui, Donglai Jing, Xuxiang Zhang, Yaojing Chen, Bingkun Li, Zhigang Liang, Kewei Chen, Zhanjun Zhang, Liyong Wu

**Affiliations:** 1 Department of Neurology, Xuanwu Hospital, Capital Medical University, Beijing, 100053 China; 2 Department of Neurology, Beijing Sixth Hospital, Beijing, 100007 China; 3 State Key Laboratory of Cognitive Neuroscience and Learning, Beijing Normal University, Beijing, 100875 China; 4 Department of Nuclear Medicine, Xuanwu Hospital, Capital Medical University, Beijing, China; 5 Department of Ophthalmology, Xuanwu Hospital, Capital Medical University, Beijing, China; 6 Banner Alzheimer’s Institute, Phoenix, AZ USA; 7 National Clinical Research Center for Geriatric Disorders, Capital Medical University, Beijing, 100053 China

**Keywords:** Alzheimer’s Disease, Agnosia, Magnetic resonance imaging, Abnormalities, Visual pathways

## Abstract

Simultanagnosia is a common symptom of posterior cortical atrophy, and its association with brain structural and functional changes remains unclear. In our study, 18 posterior cortical atrophy patients with simultanagnosia, 29 patients with Alzheimer’s disease and 20 cognitively normal controls were recruited and subjected to full neuropsychological evaluation, including simultanagnosia tests, and structural and resting-state functional MRI. The gray matter volume was assessed by voxel-based morphometry, while the intrinsic functional connectivity was evaluated using the reduced gray matter volume regions of interest as the seed. In contrast to the patients with Alzheimer’s disease, those with posterior cortical atrophy showed the following: (1) markedly lower simultanagnosia test scores, (2) an altered regional gray matter volume of the left middle occipital gyrus and ventral occipital areas, and (3) lowered intrinsic functional connectivity with the left middle occipital gyrus, left lingual gyrus and right middle occipital gyrus separately. Additionally, the gray matter volume of the left middle occipital gyrus and left inferior occipital gyrus were each correlated with simultanagnosia in posterior cortical atrophy patients. The intrinsic functional connectivity of the left middle occipital gyrus with the right superior occipital gyrus and that of the right middle occipital gyrus with the left superior parietal gyrus were also correlated with simultanagnosia in posterior cortical atrophy patients. In summary, this study indicated that simultanagnosia is associated with gray matter reductions and decreased functional connectivity in the left middle occipital gyrus and the left inferior occipital gyrus in patients with posterior cortical atrophy.

## Introduction

As observed in wounded veterans with visuospatial impairment (Hughes, 1970) and more systematically described by Benson et al. in (1988), posterior cortical atrophy (PCA) is a clinical syndrome characterized by visual impairment and the associated parietal occipital cortical atrophy, most of which have the same pathologies as Alzheimer’s disease (AD) and other syndromes such as Lewy body pathology, corticobasal degeneration, prion disease and others (Maia da Silva et al*.*, 2017; Graff-Radford et al., 2021). In the early stage of PCA, simultanagnosia is one of the most representative symptoms (Singh et al., 2015; Tang-Wai et al., 2004). Patients with simultanagnosia cannot recognize and classify objects when they appear together but can do so when each object appears separately (Crutch et al., 2011). Previous studies have hypothesized that simultanagnosia is associated with parietal lobe damage (Hughes, 1970).

Few studies have investigated the neuroimaging characteristics of patients with simultanagnosia (Kas et al., 2011; Neitzel et al., 2016; Sakurai et al., 2016, 2018). Among them, two case reports documented the specific encephalic changes in cerebral ischemia patients who had simultanagnosia using structural magnetic resonance imaging (sMRI) and single photon emission computed tomography (Sakurai et al., 2016, 2018). The authors suggested that simultanagnosia is related to the right temporoparietal area in one patient and the left parietooccipital lobe in the other (Sakurai et al., 2016, 2018). A case–control study published in 2016 comparing sMRI findings between 12 PCA subjects and 12 healthy controls reported that white matter atrophy within the left association fiber pathway results in simultanagnosia. Additionally, the effect of other cognitive impairment (such as memory, language and executive function) on the sMRI-based findings could not be assessed because of the lack of a AD group (Neitzel et al., 2016). Another study used single photon emission computed tomography (SPECT) to examine an AD group and a PCA group with matched Mini-Mental State Examination (MMSE) scores; the study found that hypoperfusion in the right inferior occipital gyrus and bilateral middle occipital gyri was related to simultanagnosia (Kas et al., 2011).

We designed the current study to systematically investigate the association of visual impairment with both structural and intrinsic functional connectivity (iFC) anomalies using sMRI and resting-state functional MRI (rs-fMRI) techniques in patients with PCA. To mitigate the influence of the severity of other cognitive dysfunction, we enrolled typical AD patients matched for disease course and clinical dementia rating (CDR) scores as well as cognitively normal controls (NCs) to characterize the unique correlate in PCA patients. Using this design, we attempted to explore the onset locus of simultanagnosia and explain its causes, providing a basis for further research on the diagnosis and treatment of this syndrome.

## Materials and Methods

### Participants

From August 2017 to June 2018, 18 patients with PCA, 29 patients with typical AD and twenty NCs were recruited by the Department of Neurology in Xuanwu Hospital. We obtained written informed consent from the individuals or their families to participate in this study according to the Declaration of Helsinki. The informed consent form was approved by the Ethics Committee of Xuanwu Hospital, Capital Medical University on Human Clinical Research.

All the participants completed detailed clinical history interviews, physical examinations and neuropsychological assessments. Each patient with PCA fulfilled the diagnostic criteria developed in 2017 (Crutch et al., 2017) and reported vision-related cognitive problems as chief complaints and early symptoms rather than memory, language or executive functions. Patients with typical AD met the diagnosis criteria established by the International Working Group (IWG-1) (Dubois et al., 2007). All the patients had a disease duration less than 5 years and a CDR score ≥ 1. None of the patients were tested for cerebrospinal fluid restricted by practical conditions, while only seven PCA patients and seven typical AD patients completed the AV45-PET test, the results of which were positive. Additionally, subjects in the NC group showed values above the cutoff for the education-adjusted MMSE (> 19 for illiterate, 22 for primary school and 24 for secondary school and above) and the Montreal Cognitive Assessment (MoCA, 13, 19 and 24 separately for illiterate, primary and secondary schools) (Folstein et al*.*, 1975; Nasreddine et al., 2005). Furthermore, all the NC subjects had 0 scores for the CDR sum of boxes (Hughes et al., 1982). As detailed below, all the subjects completed the whole neuropsychological examination and imaging examination in a month, without any other diseases, such as eyesight problems, depression, cerebrovascular disease, poisoning, infection and metabolic diseases, explaining the patient's memory impairment and related symptoms. All the subjects had received professional ophthalmic examination, and their corrected visual acuity did not affect reading.

### Neuropsychological assessment

The severity of global cognitive decline was assessed by the CDR, and general cognitive function was assessed using the Mini-Mental State Examination (MMSE) and Montreal Cognitive Assessment (MoCA). Episodic memory was evaluated by the World Health Organization University of California-Los Angeles Auditory Verbal Learning Test (AVLT), including the AVLT learning, delayed recall and cued recall subtests (Maj et al., 1993). Language ability was assessed using the Boston Naming Test (BNT), while attention and executive function were detected by the modified Trail Making Test (TMT) part A (Arbuthnott & Frank, 2000; Cheung et al., 2004; Williams et al., 1989).

Two simultanagnosia tests were used to assess the simultaneous perception of participants. The first is the classical Poppelreuter-Ghent’s Overlapping Figures Test (PGOF), which asks the subject to name each individual object that overlaps with each other in a given figure (Della Sala et al., 1995). The other method is a computer-based test designed and developed by Beijing Normal University (Pelak et al., 2011). In the computer-based test, the subjects sat in a chair with their eyes approximately 50 cm away from the computer screen on which a large geometric shape comprising several small but identical geometric shapes, including a triangle, square and circle, was randomly presented. Before the test began, patients were asked to identify the three shapes, and people who could not be identified were not tested. This test was divided into two parts: the local shape test and global shape test. In the local shape test, the participants were required to ignore the shape of the whole figure and determine the shape of the same small ones; in the global shape test, they had the opposite requirement. Each of the two parts contained 54 geometric shapes, with five trials before the test. The participants’ responses were recorded by the computer, and one point was awarded for each correct answer. Additionally, the response time was automatically recorded by the computer regardless of whether the response was correct or incorrect (see Additional Fig. 4).

Optic ataxia was assessed using the method described by Karnath and Perenin (2005). Visuospatial function was assessed by the revised Rey-Osterrieth Complex Figure Test, and a rapid automatized naming test was used to examine reading ability (Shin et al., 2006; Wang et al., 2017). Additionally, facial agnosia was evaluated by accurately naming ten celebrities (including national leaders and actors) based on images.

### MRI scanning and imaging parameters

Magnetic resonance scanning was performed using a GE Signa PET/MR 3.0 Tesla scanner (GE Healthcare, Milwaukee, WI) at Xuanwu Hospital, Capital Medical University. During the scanning, the participants were asked to remain quiet with closed eyes and to think of nothing in particular. High-resolution T1-weighted structural images and resting-state functional images were acquired from all the participants using the following parameters: structural data were collected using 3D magnetization prepared rapid gradient echo sequences (192 sagittal slices; repetition time/echo time = 6.9/3.0 ms; slice thickness = 1 mm; flip angle = 12°; acquisition matrix = 256 × 256; field of view = 256 mm × 256 mm), and functional data were obtained using an echo-planar imaging sequence over 8 min (repetition time/echo time = 2000/30 ms; flip angle = 90°; 36 slices; slice thickness = 3.5 mm; acquisition matrix = 64 × 64; field of view = 230 mm × 230 mm; 240 images). FLAIR sequences were also acquired from all the participants to exclude the presence of lesions.

### Data processing and analyses

#### Structural image preprocessing and analysis

The CAT12 toolbox (www.neuro.uni-jena.de/cat) was used to preprocess the images using all default settings. The images were bias-corrected, tissue classified, and normalized to the Montreal Neurological Institute (MNI) space at 1.5-mm isotropic voxel resolution using linear (12-parameter affine) and nonlinear transformations within a unified model including the high-dimensional Diffeomorphic Anatomical Registration Through Exponentiated Lie Algebra (DARTEL) normalization (Ashburner, 2007). Gray and white matter segments were modulated to preserve the actual tissue amount locally. After quality control (QC) and homogeneity inspections, all the images were smoothed using a Gaussian kernel of 8 mm full-width-half-maximum (FWHM). We conducted voxel-based morphometry (VBM) analyses to explore gray matter volume (GMV) differences among the PCA, typical AD and NC groups by analysis of covariance (ANCOVA) with age, sex, years of education and the total intracranial volume (TIV) as covariates, and the significance was judged when *p* < 0.05 with familywise error (FWE) correction for multiple comparisons. The mean values of significant clusters identified were then extracted for further analyses.

#### Resting-state functional image preprocessing and analysis

Images were processed using the Data Processing & Analysis for Brain Imaging toolbox (Yan et al., 2016). The first ten volumes from each scan were excluded as dummy scans to allow magnetization stability. Slice timing correction and image realignment to correct the head motion were followed; after the segmentation of coregistered T1-weighted structural images, realigned functional images were spatially normalized to the MNI space at 3 mm isotropic voxel resolution based on transformation parameters computed with the DARTEL tool. The linear trend, head motion parameter measured using the Friston-24 model, white matter, and cerebrospinal fluid signals were further regressed out as nuisance covariates (Price et al*.*, 1996). Next, the resultant images were temporally filtered (0.01–0.1 Hz) and smoothed using a 4-mm FWHM Gaussian kernel.

To inspect how group differences in GMV affect cortical functional coupling, clusters that significantly differed between typical AD and PCA in the above VBM analyses were used to construct resting-state iFC maps. Briefly, 5-mm-radius balls centered at the peak coordinates of VBM clusters were drawn and defined as seed regions, and then the raw iFC map for each seed region was constructed by extracting the mean rs-FMRI time series from each seed region and correlating them to the time series of every other voxel in the brain using Pearson’s correlation. After Fisher’s r to z transformation, the normalized iFC maps for each seed region were compared among groups by voxelwise ANCOVA in the Statistical Parametric Mapping toolbox (SPM12, https://www.fil.ion.ucl.ac.uk/), adjusted for the effects of age, gender, years of education and TIV; Monte Carlo simulation was applied to correct for multiple comparisons (*p* < 0.001 uncorrected, 10,000 iterations) to achieve a cluster-level false-positive rate of 0.05. For significant clusters found in pairwise comparisons, the mean iFC over a given cluster was extracted for subsequent correlation analyses with simultanagnosia measures within the PCA group.

### Statistical analyses

ANOVA was performed to compare demographic and neuropsychological data among the three groups in SPSS (version 22.0; IBM Inc., New York, NY). To explore the relationships between the neuroimaging findings and simultanagnosia, partial correlation analyses were performed in the PCA group between the results of simultanagnosia tests and GMV and the iFCs after regressing out covariates such as age, sex, years of education, disease duration and the TIV (for VBM findings) or GMV of seed regions (for iFC findings). A statistical significance level of *p* < 0.05 was used in these analyses.

## Results

### Demographic, neuropsychological and visuospatial assessment

No statistically significant difference was found in age, years of education or sex ratio, while significant differences were shown in the TMT A test (F = 39.44; *p* < 0.01), reading test (F = 21.26; *p* < 0.01) and facial agnosia test (F = 10.28; *p* < 0.01) among the three groups; the PCA group performed worse than the other group. Additionally, no significant difference was found in the disease duration, immediate or delayed memory and CDR scores between the PCA and typical AD groups. (t = 0.73, *p* = 0.47; t = 1.69, *p* = 0.10; t = 1.90, *p* = 0.07; t = 0.89, *p* = 0.38) (Table 1 and Additional Table 3).

**Table 1:** Demographics, neuropsychological and visuoconstruction assessment of the three groups

	PCA group (n = 18)	typical AD group (n = 29)	NC group (n = 20)	F/c 2	*p*
Sex (male/female)	7/11	12/17	13/7	3.42	0.18
Age (years)	57.56 ± 5.04	58.31 ± 5.22	54.95 ± 8.19	1.80	0.17
Years of education	9.22 ± 5.24	11.48 ± 3.38	11.50 ± 2.96	2.27	0.11
Age at diagnosis (years)	53.61 ± 4.45	54.62 ± 5.86	–	− 0.63	0.53
Disease duration (years)	3.94 ± 1.51	3.59 ± 1.70	–	0.73	0.47
*Cognitive screening test*					
MMSE (30)	13.11 ± 5.26	17.28 ± 6.85	29.30 ± 0.80	49.80	< *0.01*^a,b^
MoCA (30)	7.39 ± 3.84	12.41 ± 7.30	27.40 ± 1.60	76.82	< *0.01*^a,b^
global CDR (3)*	1.42 ± 0.55	1.26 ± 0.62	0.00 ± 0.00	49.58	< *0.01*^a^
CDR sum of boxes (18)*	8.361 ± 3.27	6.603 ± 4.09	0.00 ± 0.00	44.53	< *0.01*^a^
*Neuropsychological assessment*					
AVLT learning (45)	7.22 ± 6.50	10.35 ± 5.96	24.10 ± 5.30	46.20	< *0.01*^a^
Delayed recall (15)	0.28 ± 0.67	1.03 ± 1.97	9.25 ± 1.83	181.23	< *0.01*^a^
BNT (30)	13.94 ± 5.41	17.69 ± 6.10	25.70 ± 2.25	27.57	< *0.01*^a,b^
TMT A Time (150 s)**	150.00 ± 0.00	116.03 ± 47.07	47.65 ± 17.02	50.10	< *0.01*^a,b^
TMT A No. of correct lines (24)	2.72 ± 5.22	13.07 ± 10.40	24.00 ± 0.00	39.44	< *0.01*^a,b^
Optic ataxia test (4)	2.17 ± 1.76	3.72 ± 1.03	4.00 ± 0.00	14.63	< *0.01*^a,b^
ROCFT (16)	1.11 ± 3.18	8.03 ± 6.52	15.05 ± 0.76	43.01	< *0.01*^a,b^
Reading test (s)	84.25 ± 33.28	43.52 ± 27.35	20.59 ± 3.94	21.26	< *0.01*^a,b^
Facial agnosia (10)	6.50 ± 3.11	8.17 ± 2.66	10.00 ± 0.00	10.28	< *0.01*^a,b^

*AD* Alzheimer’s disease, *BNT* Boston Naming Test, *CDR* Clinical Dementia Rating, *MMSE* Mini-Mental State Examination, *MoCA* Montreal cognitive assessment, *NC* cognitively normal control, *PCA* posterior cortical atrophy, *ROCFT* Rey-Osterrieth Complex Figure Test, *TMT* Trail Making Test

^a^Significant when comparing both patient groups to the NC group

^b^Significant when comparing the PCA and typical AD groups

*Lowest/worst value for the assessment

**Time limit for the test. Values in parentheses not marked with an asterisk indicate the highest/best value for the assessment

The PGOF and computer-based global shape test scores were significantly different among the three groups (F = 62.33, *p* < 0.01; F = 79.82, *p* < 0.01); the PCA group showed significantly lower scores than the typical AD group (t = 7.63, *p* < 0.01; t = 9.19, *p* < 0.01), while no apparent differences were found in the computer local shape test scores among the three groups (F = 1.91, *p* = 0.16) and clinical groups (t = 1.25, *p* = 0.23). Additionally, the time of the computer-based local shape test in the PCA group was significantly longer than that of the typical AD group (t = 3.01, *p* < 0.01), while the time of the computer-based global shape test was not different (t = 0.66, *p* = 0.52) (Table 2 and Additional Table 4).

**Table 2:** Simultanagnosia test score of the three groups

	PCA group (n = 18)	Typical AD group (n = 29)	NC group (n = 20)	F	*p*
Poppelreuter-Ghent’s overlapping figures test score (4)	1.11 ± 0.68	3.10 ± 1.11	4.00 ± 0.00	62.33	< *0.01*^*a,b*^
Computer test score (global) (1)	0.43 ± 0.20	0.93 ± 0.17	0.99 ± 0.01	79.82	< *0.01*^*a,b*^
Computer test score (local) (1)	0.91 ± 0.10	0.94 ± 0.02	0.94 ± 0.02	1.91	0.16
Computer test response time (global) (s)	425.09 ± 218.13	360.11 ± 383.41	62.04 ± 13.94	9.81	< 0.01^a^
Computer test response time (local) (s)	269.24 ± 146.93	159.55 ± 102.79	55.36 ± 12.77	20.83	< 0.01^a,b^

The data are presented as means ± standard deviation

*AD* Alzheimer’s disease, *NC* cognitively normal control, *PCA* posterior cortical atrophy

^a^Significant when comparing both patient groups to the NC group

^b^Significant when comparing the PCA and typical AD groups

### Gray matter volume in the cerebral cortex

**Fig. 1:**
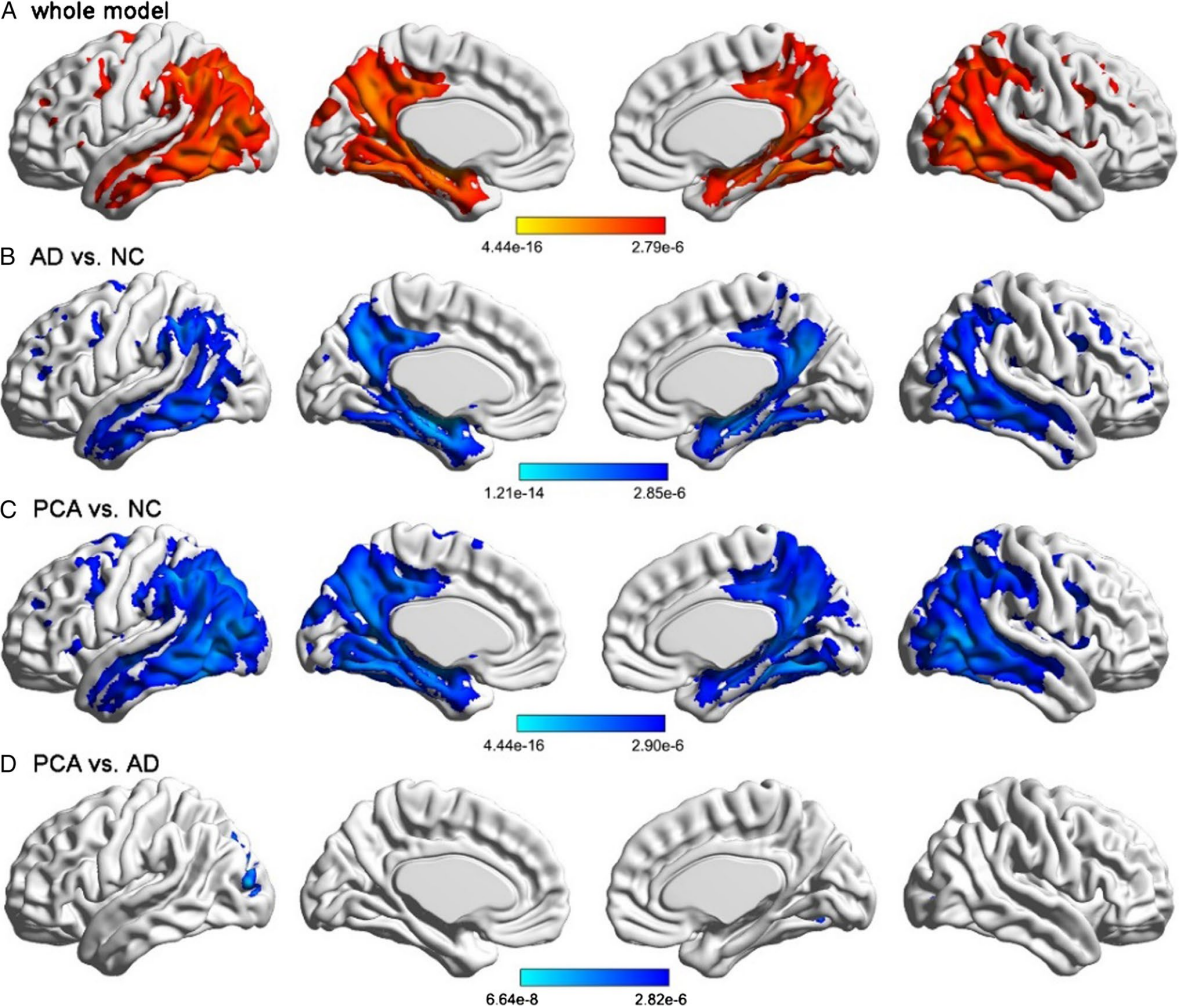
Group comparisons of the whole-brain gray matter volume. **A** Comparison of the NC, typical AD and PCA groups using ANCOVA. **B** Reduced GMV in the typical AD group relative to the NC group. Reduction was mainly found in the bilateral middle temporal lobe, precuneus, posterior cingulate cortex, and left temporoparietal cortices. **C** Reduced GMV in the PCA group relative to the NC group. Reduction was mainly found in the left superior occipital lobe, right fusiform lobe, left thalamus, left superior temporal lobe and right middle frontal lobe. **D** Reduced GMV in the PCA group relative to the typical AD group. Reduction was mainly found in the left middle occipital gyrus, along with ventral occipital areas. All the results were controlled for the effects of age, sex, years of education and TIV and survived the FWE corrected *p* < 0.05. The color bars indicate p values of the ANCOVA and post hoc comparisons among the three groups, and brighter colors represent higher significance. *NC* cognitively normal control, *AD* Alzheimer’s disease, *PCA* posterior cortical atrophy

Comparison of the NC, typical AD and PCA groups using ANCOVA is shown in Fig. 1A. As shown in Fig. 1B, C, VBM analysis revealed a typical spatial map of GMV atrophy in both typical AD and PCA patients compared with NC participants, including the bilateral middle temporal lobe, precuneus, posterior cingulate cortex, and left temporoparietal cortices, and this spatial pattern extended to the posterior occipital cortex in PCA patients (details on the cluster coordinates are provided in Additional Table 5). Notably, the brain regions surviving the FWE correction in the comparison between PCA and typical AD were located in the left middle occipital gyrus (cluster size = 1376; t = 5.980; *p* = 6.64e − 8), left lingual gyrus (cluster size = 162; t = 5.848; *p* = 1.10e − 7), right fusiform gyrus (cluster size = 116; t = 5.743; *p* = 1.65e − 7), right middle occipital gyrus (cluster size = 114; t = 5.328; *p* = 7.86e − 7), and left inferior occipital gyrus (cluster size = 99; t = 5.927; *p* = 8.14e − 8), particularly the left middle occipital gyrus and ventral occipital areas (Fig. 1D and Additional Table 6).

### Reduced functional connectivity

**Fig. 2:**
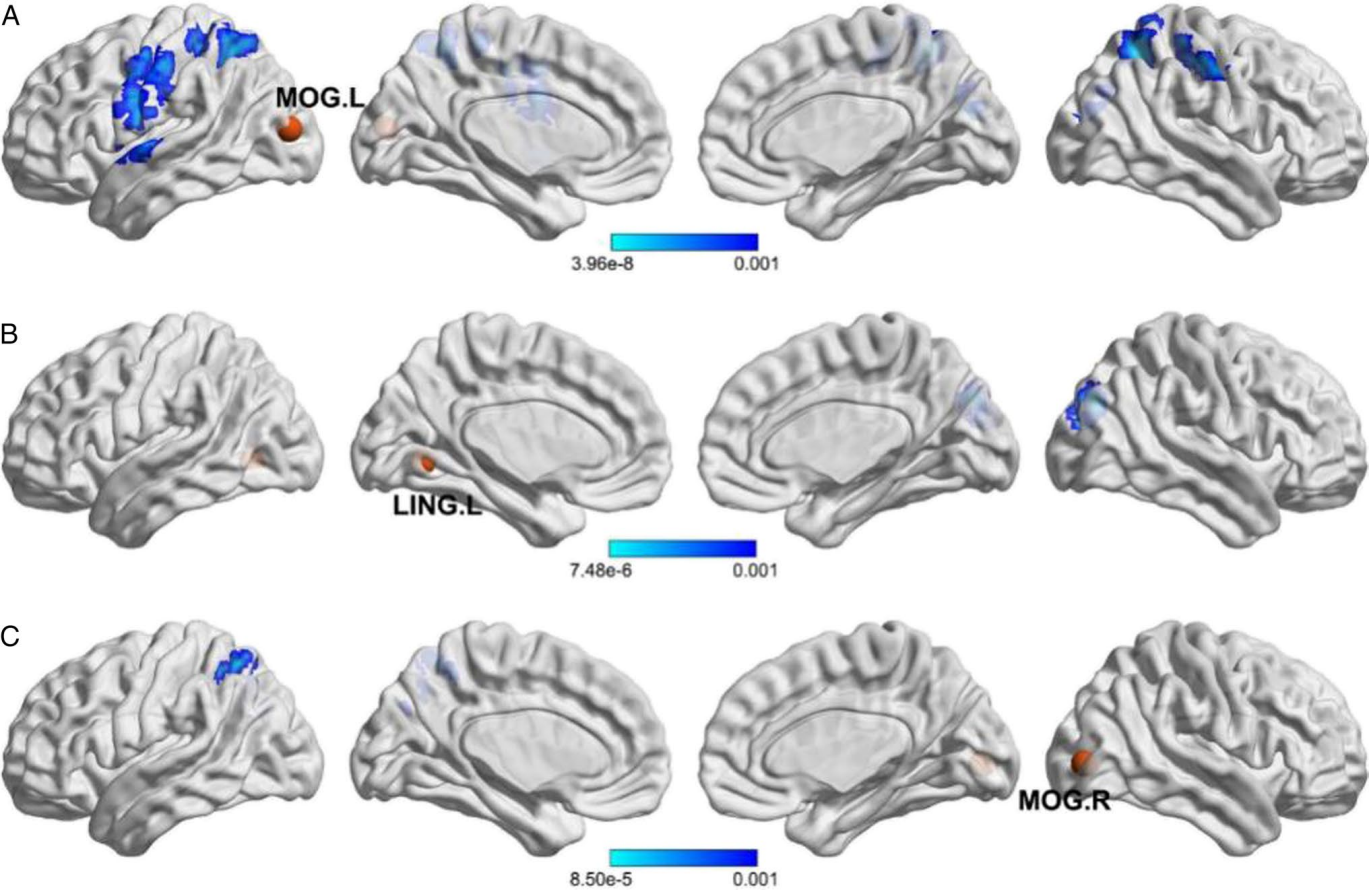
Reduced intrinsic functional connectivity in posterior cortical atrophy compared with Alzheimer’s disease. Five regions with reduced GMV in PCA were used as seed regions to construct iFC maps. Using ANCOVA models that included age, sex, years of education and TIV as covariates, comparisons showed significantly decreased iFC in PCA compared with typical AD, particularly those derived from the left middle occipital gyrus (upper row **A**), left lingual gyrus (middle row **B**) and right middle occipital gyrus (lower row **C**). All the results were thresholded at corrected *p* < 0.05 after multiple comparisons. The color bars indicate p values of the comparisons between PCA and typical AD, and brighter colors represent higher significance. *MOG. L* left middle occipital gyrus, *LING. L* left lingual gyrus, *MOG. R* right middle occipital gyrus

To determine how GMV atrophy affects cortical functional coupling, the regions with significant atrophy in the PCA group compared with those in the typical AD group were selected as seed regions (Additional Table 7). By constructing seed-based iFC maps based on rs-fMRI, voxelwise comparisons between the PCA and typical AD groups showed significantly decreased functional connectivity in PCA patients. More specifically, as shown in Fig. 2 and Additional Table 7, regions with reduced connections to the left middle occipital gyrus included the bilateral postcentral gyrus, left precentral gyrus, right superior occipital gyrus, right inferior parietal gyrus and left superior parietal gyrus, and the functional connectivity between the left lingual gyrus and right superior occipital gyrus, as well as the connectivity between the right middle occipital gyrus and left superior parietal gyrus, was more damaged in PCA patients than in typical AD patients.

### Correlation between simultanagnosia and between-group MRI differences

**Fig. 3:**
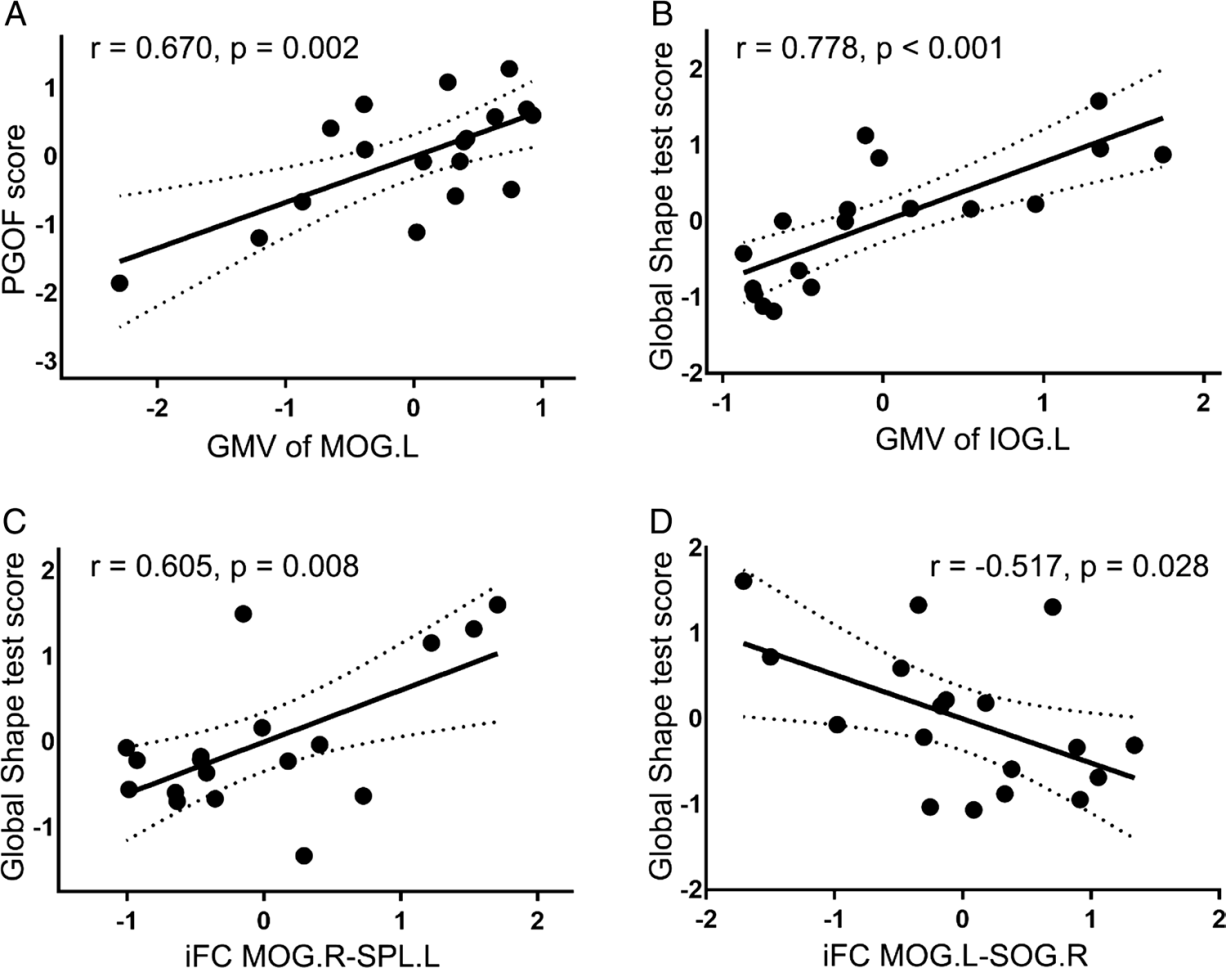
Relationships between the simultanagnosia test and gray matter volume and intrinsic functional connectivity. **A** Positive correlation between the picture test and GMV of the left middle occipital gyrus. **B** Positive correlation between the global shape test and GMV of the left inferior occipital gyrus. **C** Positive correlation of the global shape test with the iFC between the right middle occipital gyrus and left superior parietal gyrus. **D** Negative correlation of the global shape test with the iFC between the left middle occipital gyrus and right superior occipital gyrus. Partial correlations were conducted by regressing out the effects of age, gender, years of education, disease duration and TIV (for VBM findings) or GMV of seed regions (for iFC findings). *MOG. L* left middle occipital gyrus, *IOG. L* left inferior occipital gyrus, *MOG. R* right middle occipital gyrus, *SPL. L* left superior parietal gyrus, *SOG. R* right superior occipital gyrus, *PGOF* Poppelreuter-Ghent’s Overlapping Figures Test

Partial correlation analyses associated measures of simultanagnosia with GMV clusters corresponding to atrophy in the PCA group relative to the typical AD group. In PCA group, the GMV of the left middle occipital gyrus was associated with PGOF (r = 0.670, *p* = 0.002), the GMV of the left inferior occipital gyrus was correlated with the accuracy rate of the computer global shape test (r = 0.778; *p* < 0.001; Fig. 3 and Additional Table 8). Associations between PGOF and GMV clusters corresponding to atrophy in the typical AD patient relative to the NC group were also found, such as the GMV of the right hippocampus, left thalamus, right caudate and bilateral middle frontal gyrus (Additional Table 9).

Regarding iFC indices from the seed regions that GMV clusters corresponding to atrophy in the PCA group compared with those in the typical AD group, we found that in the PCA group, the iFC between the left middle occipital gyrus and right superior occipital gyrus, together with the iFC between the right middle occipital gyrus and left superior parietal gyrus, were associated with the computer global shape test score (r = − 0.517, *p* = 0.028; r = 0.605, *p* = 0.008; Fig. 3 and Additional Table 8).

## Discussion

In the present study, PCA patients were selected as the experimental group to examine the imaging abnormalities of simultanagnosia in not only brain structure but also brain iFC. The typical AD group was included in this study to specifically eliminate the impact of irrelevant variables.

Our results showed that compared with the typical AD group, reduced GMV in the PCA group occurred in five different brain regions, mainly in the left middle occipital gyrus and ventral occipital area, which were positively correlated with the scores of the PGOF or computer global shape test. Among these atrophic brain regions, only the left middle occipital gyrus, left lingual gyrus and right middle occipital gyrus showed decreased functional connections. Furthermore, we found correlations between the bilateral middle occipital gyrus and computer global shape test scores. Overall, our findings suggested that simultanagnosia is related to the left middle occipital gyrus and left inferior occipital gyrus.

### Reduced gray matter volume and intrinsic functional connectivity related to simultanagnosia in patients with posterior cortical atrophy

In our study, the PGOF and computer-based global shape test scores were adopted as the principal standards to evaluate the presence and severity of simultanagnosia, the specificity of which is worth noting, considering that PCA usually exhibits many other visual deficits except simultanagnosia concurrently.

As reported, the PGOF has been used to assess figure-ground segregation, visuospatial function and visuo-perceptual function (Glick-Shames et al., 2019). Additionally, excessive visual crowding, which disrupts nearby stimuli to central image recognition and is associated with reduced GMV within occipital regions, may interfere with the PGOF result to a large extent (Yong et al*.*, 2014). In our neuropsychological assessment, the BNT, ROCFT and reading test scores were significantly different among the three groups, suggesting that the recruited PCA patients possibly had deficits in object perception, visuospatial function and excessive visual crowding and may explain their low performance in the PGOF. To compensate for this limitation, a computer-based test was used to increase test-function correspondence and specificity for simultanagnosia.

In contrast to the PGOF, the computer-based simultanagnosia test has a low requirement on the subject's ability in object perception but focuses on the patient’s ability to recognize the global and local shape of objects. Combined with our research results, the global computer test score of PCA patients was significantly lower than that of typical AD patients, while the local computer test score showed no significant difference, indicating that the basic visual function of PCA patients was the same as that of typical AD patients but with severe simultanagnosia rather than with excessive visual crowding.

Most previous imaging studies on PCA patients did not correlate the imaging results with their clinical manifestations or merely explored the relationship between the MRI results and visual perception disorders in general, without focusing on a complex and characteristic symptom (Glick-Shames et al., 2019; Wang et al*.*, 2015; Whitwell et al., 2007). In the present study, according to the structural and rs-fMRI measures, significant differences were observed between PCA and typical AD, not only the GMV but also its decreased iFC associated with simultanagnosia in PCA patients.

Our results showed that the brain regions with significantly reduced GMV in PCA patients were the left middle occipital gyrus, whose iFC to the right superior occipital gyrus, bilateral postcentral gyrus, left precentral gyrus, right inferior parietal gyrus, left superior parietal gyrus and left superior temporal gyrus also decreased distinctly, and ventral occipital areas including the left lingual gyrus, left inferior occipital gyrus, right fusiform gyrus and right middle occipital gyrus. Additionally, both reductions were positively correlated with simultanagnosia, providing evidence that simultanagnosia is related to atrophy of the left middle occipital gyrus and left inferior occipital gyrus. Although both PCA and typical AD showed atrophy in part of the lateral posterior temporal lobe compared with normal participants, no significant atrophy was identified in PCA compared with typical AD in posterior temporal regions (Fig. 1). This finding may indicate that posterior temporal atrophy is not a general GMV feature among our PCA patients. Furthermore, we did not identify any relationships between GMV of the temporal lobe and reading ability in additional analyses in PCA patients.

Another interesting finding of our current study was the negative correlation of simultanagnosia with the iFC between the left middle occipital gyrus and right superior occipital gyrus. With the lack of other possible interpretations and need for additional studies, this finding suggests the presence of a compensatory mechanism. The increased connectivity between the right middle occipital gyrus and left superior parietal gyrus of PCA patients reinforced our conclusion. Simultanagnosia was more dependent on iFC malfunction between the occipital cortex and higher visual processing cortex and negatively correlated with functional connectivity within the occipital lobe. Additionally, the right middle occipital gyrus was atrophic in the PCA group, with a decreased iFC to the left superior parietal gyrus, consistent with the observed positive correlation with simultanagnosia. Again, because of the limited number of samples and requirement for direct hypothesis-based evidence, additional studies are needed to confirm our findings, particularly the role of the right middle occipital gyrus in simultanagnosia.

### Primary visual cortex lesions are related to simultanagnosia in patients with posterior cortical atrophy

The primary visual cortex of humans is located in the Brodmann 17 area, which is the neural basis for the earliest processing of visual stimuli and visual features, including directional movement, spatial frequency, parallax and color (Tang-Wai et al., 2004). Consistent with the findings for the primary visual cortex, our findings regarding the left middle occipital gyrus and left inferior occipital gyrus and their volumetric/iFC association with simultanagnosia suggest a reasonable hypothesis that the lesion of the primary visual cortex may be related to simultanagnosia in PCA patients.

Some studies have attributed simultanagnosia to the lesion of the unilateral region along the ventral visual pathway, and others have found an association between simultanagnosia and the dorsal pathway (Fink et al., 1996, 1997; Shames et al., 2015; Thomas et al., 2012). However, others have suggested that the change in the temporal parietal lobe, the terminal of the visual dorsal pathway, is highly correlated with the development of simultanagnosia (Himmelbach et al., 2009; Zaretskaya et al., 2013). Additionally, the hypothesis that attention deficit results in simultanagnosia remains the most widely accepted viewpoint currently, but some studies have confirmed that the attention network is largely affected by the ventral visual pathway (Chechlacz et al*.*, 2012; Dalrymple et al., 2013; Roelfsema et al., 1998). However, these mechanistic studies have ignored a key point: although the visual pathway of human beings is divided into the dorsal and ventral pathways, they both start from the primary visual cortex (Ungerleider and Haxby, 1994). Combined with our study results, we suggest that the development of simultanagnosia in PCA patients may be related to dysfunction of the visual pathway originating from the primary visual cortex.

### Clinical implications and limitations

The results of this study may help distinguish visual functional impairment characterized by simultanagnosia and other ocular dysfunctions, contributing to the diagnosis of PCA. This conclusion may also provide a target cerebral region for further studies on the treatment of patients with simultanagnosia. Thus, we are exploring the therapeutic effects of different targets under transcranial alternating current stimulation in AD and PCA patients (Ritzinger et al*.*, 2012). Overall, the greatest limitation of our study is the relatively small sample size. Additionally, although strict diagnostic criteria were applied to recruit PCA patients and typical AD patients, most were not tested for cerebrospinal fluid, AV45-PET was restricted by practical conditions, and amyloidosis was not part of the diagnosis. It’s worth noting that although all participants were required to have a CDR ≥ 1 and a disease duration of less than 5 years in order to match disease severity, the score of MMSE and MoCA suggested that the PCA group is more impaired than the typical AD group, which may be a source of bias. In future studies, we will increase the sample size, assess amyloidosis and conduct longitudinal follow-up to further confirm our conclusion and hypothesis.

## Conclusion

This study indicated that simultanagnosia is associated with gray matter reductions and decreased functional connectivity in the left middle occipital gyrus and left inferior occipital gyrus in PCA.

## Figures and Tables

**Fig. 4:**
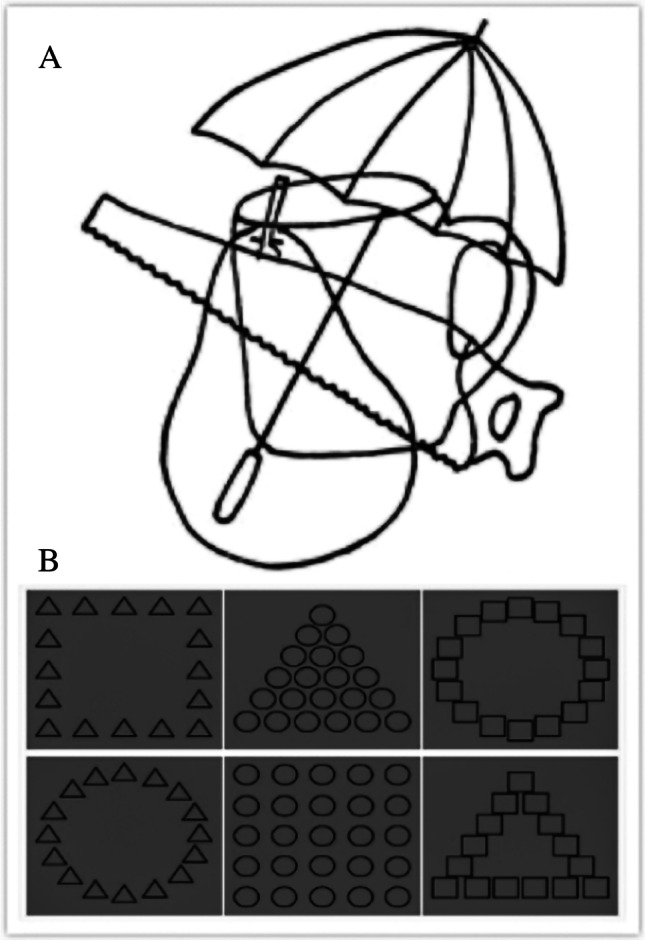
**A** Poppelreuter-Ghent’s Overlapping Figures Test (Della Sala et al., 1995); **B** computer-based test

## Data Availability

The datasets used and/or analyzed during the current study are available from the corresponding author upon request.

## Appendix

See Fig. 4 and Tables 3, 4, 5, 6, 7, 8, 9

**Table 3:** Demographics, neuropsychological and visuospatial construction assessment of the PCA and typical AD groups

	PCA group (n = 18)	typical AD group (n = 29)	t/χ2	*p*
Sex (male/female)	7/11	12/17	0.87	0.56
Age (years)	57.56 ± 5.04	58.31 ± 5.22	0.49	0.63
Years of education	9.22 ± 5.24	11.48 ± 3.38	1.63	0.12
Age of diagnosis (years)	53.61 ± 4.45	54.62 ± 5.86	0.63	0.53
Disease duration (years)	3.94 ± 1.51	3.59 ± 1.70	0.73	0.47
*Cognitive screening test*				
MMSE (30)	13.11 ± 5.26	17.28 ± 6.85	2.21	0.03
MoCA (30)	7.39 ± 3.84	12.41 ± 7.30	3.09	< 0.01
CDR (3)*	1.42 ± 0.55	1.26 ± 0.62	0.89	0.38
*Neuropsychological assessment*				
AVLT learning (45)	7.22 ± 6.50	10.35 ± 5.96	1.69	0.10
Delayed recall (15)	0.28 ± 0.67	1.03 ± 1.97	1.90	0.07
BNT (30)	13.94 ± 5.41	17.69 ± 6.10	2.14	0.04
TMT A time (150 s)**	150.00 ± 0.00	116.03 ± 47.07	3.89	< 0.01
TMT A no. of correct lines (24)	2.72 ± 5.22	13.07 ± 10.40	4.52	< 0.01
Optic ataxia test (4)	2.17 ± 1.76	3.72 ± 1.03	3.41	< 0.01
ROCFT (16)	1.11 ± 3.18	8.03 ± 6.52	4.87	< 0.01
Reading test (s)	84.25 ± 33.28	43.52 ± 27.35	3.03	< 0.01
Facial agnosia(10)	6.50 ± 3.11	8.17 ± 2.66	1.96	0.05

*AD* Alzheimer’s disease, *BNT* Boston Naming Test, *CDR* Clinical Dementia Rating, *MMSE* Mini Mental State Examination, *MoCA* Montreal Cognitive Assessment, *NC* cognitively normal control, *PCA* posterior cortical atrophy, *ROCFT* Rey-Osterrieth Complex Figure Test, *TMT* Trail Making Test

*Lowest/worst value for the assessment

**Time limit for the test. Values in parentheses not marked with an asterisk indicate the highest/best value for the assessment

**Table 4:** Simultanagnosia test of the PCA and typical AD groups

	PCA group (n = 18)	typical AD group (n = 29)	t	*p*
Poppelreuter-Ghent’s overlapping figures test score (4)	1.11 ± 0.68	3.10 ± 1.11	7.63	< 0.01
Computer test score (global) (1)	0.43 ± 0.20	0.93 ± 0.17	9.19	< 0.01
Computer test score (local) (1)	0.91 ± 0.10	0.94 ± 0.02	1.25	0.23
Computer test response time (global) (s)	425.09 ± 218.13	360.11 ± 383.41	0.66	0.52
Computer test response time (local) (s)	269.24 ± 146.93	159.55 ± 102.79	3.01	< 0.01

*AD* Alzheimer’s disease, *PCA* posterior cortical atrophy

**Table 5:** Atrophied foci in clinical patients compared with those in normal elderly patients

	MNI coordinate			Cluster size	T	*p*
	x	y	z			
*Typical AD vs. NC*						
Right hippocampus	20	− 36	8	98049	9.98	1.21e − 14
Left middle frontal gyrus	− 29	5	50	1781	7.13	7.56e − 10
Right middle frontal gyrus	30	24	42	2559	7.04	1.06e − 9
Right middle frontal gyrus	30	53	3	1785	6.68	4.45e − 9
Left middle frontal gyrus	− 15	42	35	2181	6.60	5.90e − 9
Left middle frontal gyrus, orbital part	− 24	41	− 9	754	6.56	7.10e − 9
Left thalamus	− 14	− 15	20	208	6.26	2.27e − 8
Right caudate	15	− 15	21	87	6.16	3.29e − 8
Right lingual gyrus	14	− 84	− 5	164	6.15	3.43e − 8
Left superior frontal gyrus	− 18	30	53	297	5.63	2.48e − 7
Right superior frontal gyrus, orbital part	20	24	− 14	96	5.31	8.27e − 7
*PCA vs. NC*						
Left superior occipital gyrus	− 23	− 74	24	158124	12.47	4.44e − 16
Right caudate	18	− 15	20	161	7.14	7.29e − 10
Right inferior frontal gyrus, opercular part	39	14	32	2627	6.93	1.62e − 9
Left superior temporal gyrus	− 57	− 6	6	172	5.92	8.45e − 8
Right middle frontal gyrus	29	50	18	296	5.60	2.83e − 7
Right middle frontal gyrus	32	54	2	115	5.35	7.18e − 7

The results were adjusted for the effects of age, gender, years of education and TIV and were FWE corrected *p* < 0.05 for multiple comparisons

*AD* Alzheimer’s disease, *PCA* posterior cortical atrophy, *NC* normal control

**Table 6:** Atrophied foci identified in the PCA group compared with those in the typical AD group

	MNI coordinate			Cluster size	T	*p*
	x	y	z			
Left middle occipital gyrus	− 34.5	− 85.5	12	1376	5.98	6.64e − 8
Left lingual gyrus	− 18	− 66	1.5	162	5.85	1.10e − 7
Right fusiform gyrus	28.5	− 64.5	− 9	116	5.74	1.65e − 7
Right middle occipital gyrus	36	− 84	1.5	114	5.33	7.86e − 7
Left inferior occipital gyrus	− 28.5	− 82.5	− 10.5	99	5.93	8.14e − 8

The results were adjusted for the effects of age, gender, years of education and TIV and were FWE corrected *p* < 0.05 for multiple comparisons

*AD* Alzheimer’s disease, *PCA* posterior cortical atrophy

**Table 7:** Reduced functional connectivity in PCA versus typical AD

Functional connected regions	MNI coordinate			Cluster size	T	*p*
	x	y	z			
*Seed region: left middle occipital gyrus*						
Right inferior parietal gyrus	27	− 54	54	131	6.11	3.96e − 8
Left precentral gyrus	− 48	− 3	45	202	4.78	5.91e − 6
Left superior temporal gyrus	− 54	− 9	0	51	4.70	7.88e − 6
Left superior parietal gyrus	− 27	− 57	54	83	4.66	9.06e − 6
Right postcentral gyrus	54	− 21	48	75	4.30	3.16e − 5
Right superior occipital gyrus	27	− 75	33	49	4.24	3.91e − 5
Left postcentral gyrus	− 36	− 36	57	63	4.23	4.07e − 5
*Seed region: left lingual gyrus*						
Right superior occipital gyrus	24	− 81	33	102	4.71	7.48e − 6
*Seed region: right middle occipital gyrus*						
Left superior parietal gyrus	− 30	− 60	57	74	4.01	8.50e − 5

The results were adjusted for the effects of age, gender, years of education and TIV. Monte Carlo simulation was applied for multiple comparison correction (*p* < 0.001 uncorrected, 10,000 iterations) to achieve a cluster-level false positive rate of 0.05

*AD* Alzheimer’s disease, *PCA* posterior cortical atrophy

**Table 8:** Relationships between atrophic regions in the PCA group compared with the typical AD group and simultanagnosia

	PGOF test		Global shape test	
	PCA	Typical AD	PCA	Typical AD
*Gray matter volume*				
Left middle occipital gyrus (− 34.5, − 85.5, 12)	0.670 (0.002)	0.514 (0.010)	0.398 (0.179)	0.360 (0.084)
Left lingual gyrus (− 18, − 66, 1.5)	0.185 (0.546)	0.534 (0.007)	0.542 (0.055)	0.417 (0.043)
Right fusiform gyrus (28.5 − 64.5, − 9)	0.209 (0.494)	0.433 (0.035)	0.047 (0.879)	0.408 (0.048)
Right middle occipital gyrus (36, − 84, 1.5)	0.314 (0.297)	0.352 (0.091)	0.323 (0.282)	0.323 (0.124)
Left inferior occipital gyrus (− 28.5, − 82.5, − 10.5)	0.054 (0.862)	0.464 (0.022)	0.778 (< 0.001)	0.325 (0.121)
*Intrinsic functional connectivity (seed: left middle occipital gyrus)*				
Right inferior parietal gyrus (27, − 54, 54)	− 0.209 (0.492)	0.240 (0.259)	− 0.363 (0.223)	0.369 (0.076)
Left precentral gyrus (− 48, − 3, 45)	0.021 (0.946)	0.304 (0.149)	0.193 (0.528)	0.311 (0.139)
Left superior temporal gyrus (− 54, − 9, 0)	− 0.264 (0.384)	0.187 (0.382)	− 0.068 (0.825)	0.077 (0.721)
Left superior parietal gyrus (− 27, − 57, 54)	0.032 (0.916)	0.250 (0.239)	0.147 (0.633)	0.317 (0.132)
Right postcentral gyrus (54, − 21, 48)	− 0.022 (0.942)	0.334 (0.111)	− 0.137 (0.656)	0.292 (0.166)
Right superior occipital gyrus (27, − 75, 33)	− 0.113 (0.713)	0.322 (0.125)	− 0.517 (0.028)	0.522 (0.009)
Left postcentral gyrus (− 36, − 36, 57)	− 0.145 (0.637)	0.069 (0.747)	− 0.049 (0.873)	0.278 (0.188)
*Intrinsic functional connectivity (seed: left lingual gyrus)*				
Right superior occipital gyrus (24, − 81, 33)	− 0.150 (0.624)	0.349 (0.094))	− 0.349 (0.243)	0.295 (0.162)
*Intrinsic functional connectivity (seed: right middle occipital gyrus)*				
Left superior parietal gyrus (− 30, − 60, 57)	0.303 (0.314)	0.544 (0.006)	0.605 (0.008)	0.162 (0.449)

Correlation parameters and *p* values are presented. Partial correlation analyses were performed using age, sex, years of education, total intracranial volume and disease duration as covariates

*PGOF* Poppelreuter-Ghent’s Overlapping Figures Test, *PCA* posterior cortical atrophy, *AD* Alzheimer’s disease

**Table 9:** Relationships between atrophic regions in the typical AD group compared with the NC group and simultanagnosia

	PGOF test		Global shape test	
	Typical AD	PCA	Typical AD	PCA
*Atrophic regions in typical AD compared with CN*				
Right hippocampus (20, − 36, 8)	0.463 (0.023)	0.629 (0.021)	0.462 (0.023)	0.291 (0.335)
Left middle frontal gyrus (− 29, 5, 50)	0.414 (0.044)	0.372 (0.211)	0.237 (0.264)	0.344 (0.250)
Right middle frontal gyrus (30, 24, 42)	0.292 (0.166)	0.442 (0.131)	0.197 (0.355)	− 0.158 (0.605)
Right middle frontal gyrus (30, 53, 3)	0.302 (0.152)	0.460 (0.113)	0.427 (0.037)	0.008 (0.979)
Left middle frontal gyrus (− 15, 42, 35)	0.394 (0.057)	0.489 (0.090)	0.523 (0.009)	0.300 (0.319)
Left middle frontal gyrus, orbital part (− 24, 41, − 9)	0.264 (0.212)	0.173 (0.573)	0.384 (0.064)	0.112 (0.715)
Left thalamus (− 14, − 15, 20)	0.385 (0.063)	0.140 (0.649)	0.523 (0.009)	0.313 (0.298)
Right caudate (15, − 15, 21)	0.317 (0.132)	0.155 (0.613)	0.441 (0.031)	0.126 (0.682)
Right lingual gyrus (14, − 84, − 5)	0.300 (0.154)	0.173 (0.572)	0.031 (0.887)	0.175 (0.568)
Left superior frontal gyrus (− 18, 30, 53)	0.308 (0.143)	0.321 (0.285)	0.343 (0.101)	0.220 (0.471)
Right superior frontal gyrus, orbital part (20, 24, − 14)	0.315 (0.134)	0.225 (0.460)	0.331 (0.114)	0.120 (0.695)

Correlation parameters and *p* values are presented. Partial correlation analyses were performed using age, sex, years of education, total intracranial volume and disease duration as covariates

*PGOF* Poppelreuter-Ghent’s Overlapping Figures Test, *PCA* posterior cortical atrophy, *AD* Alzheimer’s disease
